# Foreign body of the rectum: An unusual case

**DOI:** 10.4103/0974-2700.66542

**Published:** 2010

**Authors:** Y Narjis, K Rabbani, K Hakkou, T Aboulhassan, A Louzi, R Benelkhayat, B Finech, A EL Idrissi Dafali

**Affiliations:** Department of General Surgery, Marrakech, Morocco; 1 Department of Anesthesia Reanimation, CHU Mohammed VI, Cadi Ayyad University, Marrakech, Morocco

Sir,

Foreign body within the rectum occurs infrequently. Majority of objects are introduced through anus; however, sometimes a foreign body is swallowed, passes through the gastrointestinal tract and is held up in rectum. Many endoscopic and surgical techniques to remove rectal foreign bodies have been described in the literature and the reported variety of foreign bodies is as large as the number of techniques used to remove them.[1,2]

**Figure 1:**
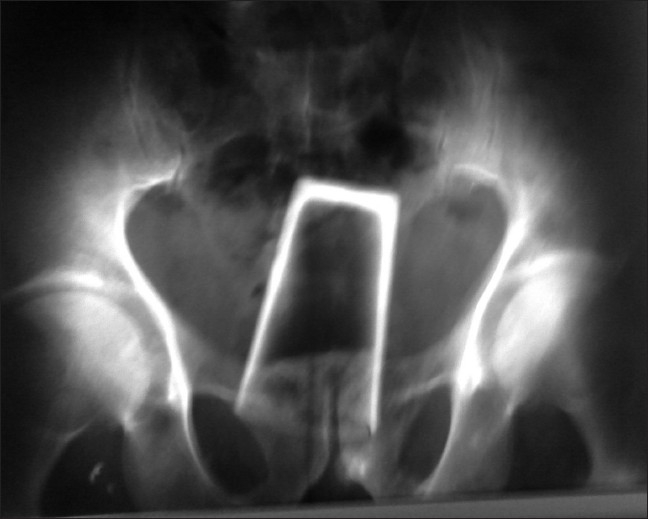
X-ray pelvis showing the glass in lower abdomen and pelvis

A 40-year-old male consulted the emergency department for sexual aggression. The patient said that he had been assaulted by two intoxicated individuals. Tying him up, they had conducted a sexual assault by forced introduction of a foreign body in the rectum. The patient had consulted 2 hours after the attack. At examination, vital signs were normal. Abdomen was soft. Foreign body was palpable on rectal examination. X-ray of the pelvis showed the glass in lower abdomen and pelvis [Figure 1].

The manual removal by holding the base of the glass was impossible and snares repeatedly slipped due to mucous coating the surface. Moreover, the glass could not be manipulated.

After reassurance and administration of IV analgesic, in lithotomy position several uni- and bi-manual extractions using obstetrics forceps had been attempted with no success and had even led to a partial fracture of the glass, making the transanal extraction extremely dangerous. Endoscopy and laparoscopy were not available in emergency. Then we proceeded to a general anesthetic and a Midline LAPAROTOMY was performed. We gradually tipped the glass to the sigmoid loop before making a short colostomy to extract the glass from sigmoid colon. A colostomy at the left iliac fossa was made. The post operative curse was uneventful and the patient left the hospital on the fourth day. The intestinal continuity was restored 3 months later.

Reports of foreign body within the rectum are uncommon in Africa, and the majority of case series are reported from Eastern Europe.[1–6] Males are commonly affected.[1,2] The age group is 16–80 years.[1] However, there is a bimodal age distribution, observed in the twenties for anal erotism or forced introduction through anus, and in the sixties mainly for prostatic massage and breaking fecal impactions.

The foreign bodies commonly reported are plastic or glass bottles, cucumbers, carrots, wooden, or rubber objects.[2] Other objects reported are bulb, tube light, axe handle, broomstick, vibrators, etc. The object length varies between 6 and 15 cm, and larger objects were more prone for complications.[2]

Abdominal and rectal pains and bleeding per rectum are the common presenting symptoms. Per rectal examination is the cornerstone in the diagnosis, but it should be performed after X-ray of the abdomen to prevent accidental injury to the surgeon from sharp objects.[4]

The first step in the evaluation is that one should always be aware of the possibility of a large bowel perforation and perform radiological investigations. Plain abdominal radiography or water soluble contrast enemas may be helpful. An abdominal X-ray will also provide information on the localization of the foreign body, whether it is below or above the rectosigmoid junction. If perforation of the bowel has occurred, immediate laparotomy is warranted. If there are no signs of perforation, several management approaches can be tried.

First, digital removal of the object should be attempted, if necessary, with the patient at different positions. If this approach fails, one can try bimanual manipulation, as we tried in our patient. The next step is the insertion of an endoscope with subsequent attempts to grasp the foreign body with regular endoscopy accessories like polypectomy snares. When this fails, it may be helpful to use devices that can be inflated in the rectosigmoid, such as a Foley catheter or an achalasia balloon. Such a device prevents a vacuum that might develop upon extraction of the foreign body and may also be directly used to remove the object.[5]

Laparotomy is only required in impacted foreign body (as in our patient) and or with perforation peritonitis. Even with laparotomy, the aim is transanal removal and closure of perforation with diversion colostomy. Post retrieval colonoscopy is mandatory to rule out colorectal injury.[6]

In conclusion, many techniques are available for the extraction of rectal foreign bodies. If possible, patients should be treated with minimally invasive techniques. When these techniques are not available or cannot extract the foreign body, surgery is required.
